# Supplementary material for: An open-source framework for large-scale, flexible evaluation of biomedical text mining systems
Source: J Biomed Discov Collab. 2008 Jan 29;3:1. doi: 10.1186/1747-5333-3-1 (PMC2276192; doi:10.1186/1747-5333-3-1)
Supplement: Additional file 1 — Evaluation platform source code. The additional file contains the software evaluation framework discussed in this paper. The most current release can be downloaded from SourceForge.net [1]. See the accompanying README file for instructions on installation and use. Also included in the distribution is a collection of UIMA wrappers for some commonly used BioNLP tools and annotated corpora. [file 1747-5333-3-1-S1.zip › bionlp_uima_repository-1.0.1/docs/api/edu/uchsc/ccp/util/nlp/parser/corpus/IProLinkCorpus1Parser.html]

IProLinkCorpus1Parser (Center for Computational Pharmacology Util/UIMA API)


|  |  |  |
| --- | --- | --- |
| **Overview** \| **Package** \| **Class** \| **Tree** \| **Deprecated** \| **Index** \| **Help** | |  |
| **PREV CLASS**   **NEXT CLASS** | **FRAMES**    **NO FRAMES**     **All Classes** |  |
| SUMMARY: NESTED \| FIELD \| CONSTR \| METHOD | DETAIL: FIELD \| CONSTR \| METHOD |  |


---


## edu.uchsc.ccp.util.nlp.parser.corpus Class IProLinkCorpus1Parser

```
java.lang.Object
  edu.uchsc.ccp.util.nlp.parser.corpus.CorpusParser
      edu.uchsc.ccp.util.nlp.parser.corpus.IProLinkCorpus1Parser
```

**All Implemented Interfaces:**: Iterator

---

``` public class IProLinkCorpus1Parser extends CorpusParser ```

A parser for the iProLink corpus1.xml file. This class outputs the original text and accompanying annotations for each corpus document. Titles and abstracts
are outputted as separate documents.  
  
Output Classes:  

- protein
- compound protein
- acronym

**Author:**
:   williamb

---

| **Field Summary** | |
| --- | --- |
| `static int` | `DOCUMENT_COLLECTION_ID` |


**Constructor Summary**

`IProLinkCorpus1Parser(String corpus1XMLFile)`


| **Method Summary** | |
| --- | --- |
| `void` | `close()` |
| `boolean` | `hasNext()` |
| `boolean` | `hasNextDocument()`             corpus1.xml has tags: protein, acronym, and compound-protein |
| `GenericDocument` | `next()` |

**Methods inherited from class edu.uchsc.ccp.util.nlp.parser.corpus.CorpusParser**

`createTextAnnotation, error, remove, warn`

**Methods inherited from class java.lang.Object**

`clone, equals, finalize, getClass, hashCode, notify, notifyAll, toString, wait, wait, wait`

**Field Detail**

### DOCUMENT\_COLLECTION\_ID

```
public static final int DOCUMENT_COLLECTION_ID
```

**See Also:**: Constant Field Values


**Constructor Detail**

### IProLinkCorpus1Parser

```
public IProLinkCorpus1Parser(String corpus1XMLFile)
                      throws IOException
```

**Throws:**: `IOException`


**Method Detail**

### close

```
public void close()
           throws IOException
```

:   **Specified by:**: `close` in class `CorpusParser`
:   **Throws:**: `IOException`

---


### hasNext

```
public boolean hasNext()
```

:   **Specified by:**: `hasNext` in interface `Iterator` **Specified by:**: `hasNext` in class `CorpusParser`

---


### next

```
public GenericDocument next()
```

:   **Specified by:**: `next` in interface `Iterator` **Specified by:**: `next` in class `CorpusParser`

---


### hasNextDocument

```
public boolean hasNextDocument()
                        throws IOException
```

:   corpus1.xml has tags: protein, acronym, and compound-protein

    :   **Parameters:**: `iProLinkCorpus1File` -: `connectionPropertiesFile` - **Throws:**: `IOException`


---


|  |  |  |
| --- | --- | --- |
| **Overview** \| **Package** \| **Class** \| **Tree** \| **Deprecated** \| **Index** \| **Help** | |  |
| **PREV CLASS**   **NEXT CLASS** | **FRAMES**    **NO FRAMES**     **All Classes** |  |
| SUMMARY: NESTED \| FIELD \| CONSTR \| METHOD | DETAIL: FIELD \| CONSTR \| METHOD |  |


---
